# Experience of road and other trauma by the opiate dependent patient: a survey report

**DOI:** 10.1186/1747-597X-3-10

**Published:** 2008-05-03

**Authors:** Albert S Reece

**Affiliations:** 1 Southcity Family Medical Centre, 39 Gladstone Rd., Highgate Hill, Queensland, Australia; 2 Medical School, University of Queensland, Herston Rd., Herston, Brisbane, Queensland, Australia

## Abstract

**Background:**

Trauma plays an important role in the experience of many patients with substance use disorder, but is relatively under-studied particularly in Australia. The present survey examined the lifetime prevalence of various forms of trauma including driving careers in the context of relevant medical conditions.

**Methods:**

A survey was undertaken in a family medicine practice with a special interest in addiction medicine in Brisbane, Australia.

**Results:**

Of 350 patients surveyed, 220 were substance dependent, and 130 were general medical patients. Addicted patients were younger (mean ± S.D. 33.72 ± 8.14 vs. 44.24 ± 16.91 years, P < 0.0001) and had shorter driving histories (15.96 ± 8.50 vs. 25.54 ± 15.03 years, P < 0.0001). They had less driving related medical problems (vision, spectacle use, diabetes) but more fractures, surgical operations, dental trauma and assaults. Addicted patients also had significantly worse driving histories on most parameters measured including percent with driving suspensions (O.R. = 7.70, C.I. 4.38–13.63), duration of suspensions (1.71 ± 3.60 vs. 0.11 ± 0.31 years, P < 0.0001), number of motor vehicle collisions (2.00 ± 3.30 vs. 1.10 ± 1.32, P = 0.01), numbers of cars repaired (1.73 ± 3.59 vs. 1.08 ± 1.60, P = 0.042), rear end collisions (O.R. = 1.90, CI 1.13–3.25), running away after car crashes (O.R. = 26.37, CI 4.31–1077.48), other people hospitalized (O.R. = 2.00, C.I. 0.93–4.37, P = 0.037) and people killed (17 vs. 0 P = 0.0005). Upon multivariate analysis group membership was shown to be a significant determinant of both cars repaired and cars hit when controlled for length of driving history. Hence use of all types of drugs (O.R. = 10.07, C.I. 8.80–14.72) was more common in addicted patients as were general (O.R. = 3.64, C.I. 2.99–4.80) and road (O.R.= 2.73, C.I. 2.36–3.15) trauma.

**Conclusion:**

This study shows that despite shorter driving histories, addicted patients have worse driving careers and general trauma experience than the comparison group which is not explained by associated medical conditions. Trauma is relevant to addiction management at both the patient and policy levels. Substance dependence policies which focus largely on prevention of virus transmission likely have too narrow a public health focus, and tend to engender an unrealistically simplistic and trivialized view of the addiction syndrome. Reduction of drug driving and drug related trauma likely require policies which reduce drug use *per se*, and are not limited to harm reduction measures alone.

## Background

From a phenomenological and pathophysiological perspective the substance dependent lifestyle is a complex mix of many factors including chemical, nutritional, genetic, judicial, congenital, social, familial and environmental factors. Trauma is one element which is often overlooked [1] and tends to compound and exacerbate many of the other aspects of this complex lifestyle. Trauma has the advantage of being relatively easy to study retrospectively by formalized questionnaire.

The attention of this clinic was drawn to the contribution of trauma in our patients' lives principally by two sets of circumstances. For some time the appalling state of our patient's teeth has been of major concern, from the point of view of aesthetic aspects, secondary nutritional compromise, the high level of associated loco-regional complications by way of the frequent dental abscesses, and also for possibly potentiating distant disease by systemic immune stimulation as has been suggested for some time by the National Institute for Dental and Craniofacial Research [2,3] and others [4,5]. A previous report from this clinic had identified that trauma was an important factor in serious dental loss [6]. This suggested that dental trauma may be associated with other kinds of trauma in our patients' lives which might contribute to a better understanding of the clinical syndrome of substance dependence. Secondly many patients have been mentioning during the course of their clinical consultations that they had been involved in motor vehicle accidents. Curiously, this was usually done in a fairly off handed way. My interest having been aroused and after making further enquiries it appeared that a frequent mechanism of accident involved collisions often into the back of another vehicle. The predominant pattern appeared to be that general medical patients had someone run into the back of their vehicle, whilst the substance dependent patients appeared to be running into the rear of cars often parked at traffic lights. Hence issues of driver inattention or mental compromise appeared to be potentially operative.

Motor vehicle trauma has been reported to be associated with over 300,000 deaths annually on a global scale [7]. Up to 25% of accidents involve drug affected drivers, and the commonest agent implicated is frequently cannabis which is said to be implicated in up to 32% – 46% of cases [6,7]. The population frequency of drug driving has been found to be about 4% in both Australia and the USA [7]. Cannabis has been reported as featuring prominently in drug driving incidents in many nations including Sweden[8], Spain [9], Italy [10], Netherlands [11], France [12-14], USA [15-17], Australia [7,18-21], and from multinational collaborations [19]. Poor driving performance including drug affected driving has been linked with deviant attitudes in other dimensions [22-24] and particularly perceived risks of detection [25-27]. Literature reports exist of impaired driver performance by opiate maintained patients treated with methadone in association with both peak and trough levels of methadone [28] and by both prescribed and illicit supplementary drugs [29,30].

The author runs a family medical clinic in Brisbane, Australia, with a special interest in the management of chemical addictions. This exposure therefore furnishes access to both substance use disorder (SUD) and general medical (N-SUD) patients and this seemed to provide an ideal opportunity to compare an SUD group with a convenience comparison sample group. Having completed and analyzed the cross-sectional survey these initial impressions have been largely confirmed. Indeed the very large degree to which many of the relevant pathologies were elevated amongst the SUD group had not been foreseen. As such these findings carry major implications for drug policy implementation in view of the inordinate peril to which the general public is exposed during the course of SUD patients procuring, using and travelling to and from their point of drug purchase [21,31]. This has ramifications for drug policy administration in that any policy which increases drug use must necessarily incur unavoidable public risk associated with drug users' access issues, and also for programs such as random drug driver screening which are increasingly generating interest in many nations [18,20,32-35]. In particular it suggests that policies such as harm reduction as it is usually framed which condone drug use *per se *[36] and focus unduly on the limitation of the spread of blood borne viruses (BBV's) [36,37], are too narrow in their purview and tend to promote a picture of substance dependence which is unrealistically benign. The marked extent to which the odds ratios of driving related trauma are elevated in SUD groups, together with the clear association with other forms of trauma imply that drug use itself must be curtailed to control these problems. Of interest these findings have come at a time when Queensland has introduced a drug driver screening program beginning in December 2007.

## Methods

### Patient recruitment

Consecutive patients presenting to our clinic were asked by the secretarial staff to complete the survey at the time of their presentation to the clinic. The survey was self administered. Patients were not paid for their participation in the study. There were no exclusions, except that the survey was aimed at present or past drivers of cars or riders of motorcycles.

### Survey completion and analysis

The surveys were completed unsupervised by patients in hardcopy. Results were then entered into a Microsoft Excel spreadsheet for data analysis.

### Clinical activity summaries

Statements of clinical activity are provided by Queensland Health from their database to practitioners upon request on an approximately annual basis. Information from such statistical summaries is included as appropriate.

### Statistics

Categorical data were analyzed, and significance levels, odds ratios and confidence intervals determined by the EpiInfo program from the Centres for Disease Control Atlanta, Georgia. Where a numerator was zero, a correction to unity was introduced to allow calculation of an odds ratio [38] as indicated in the text. Fisher exact test with two tails was used where numbers in 2 × 2 tables were less than 5. Standard adjustments to the t-test degrees of freedom (df) were applied when variances appeared unequal. This resulted in decimal values for the df. Continuous variables which were normally distributed were analyzed by a two tailed Student's t-test with different variances in the statistical software program "Statistica" from Statsoft, Tulsa, Oklahoma. Continuous variables which were not normally distributed, particularly motor vehicle trauma were compared with the non-parametric Mann-Whitney test. "Statistica" was used to prepare graphs and for multivariate analysis. Square root transforms were applied to non-normally distributed vehicular trauma data prior to multiple regression procedures. Multivariate regression was performed in the General Regression Module of Statistica on a personal computer running a Windows-XP platform. P < 0.05 was considered significant.

Patient consent, confidentiality and ethical review. All patients consented to be involved in this study. Strict patient confidentiality was assured at all times. Patient names were not collected at any stage. The study was reviewed by the Human Research Ethics Committee (HREC) of the Southcity Family Medical Centre which is a National Health and Medical Research Committee registered HREC. This study was in compliance with the Declaration of Helsinki for human experimentation.

## Results

The survey was undertaken over two weeks in October 2007. 350 patients completed the survey. During that time we performed 660 consultations including repeat visits. However the exact survey completion rate is not known. It is however likely to be of the order of 350/660 = 53.0%.

Queensland Health statistics show that this clinic has been responsible for 3,518 of 5,539 (63.5%) registered buprenorphine withdrawal patients in Queensland 2001–2006, and for 8,044 of 10,987 (73.2%) episodes of buprenorphine withdrawal in that same period.

**Figure 1:**
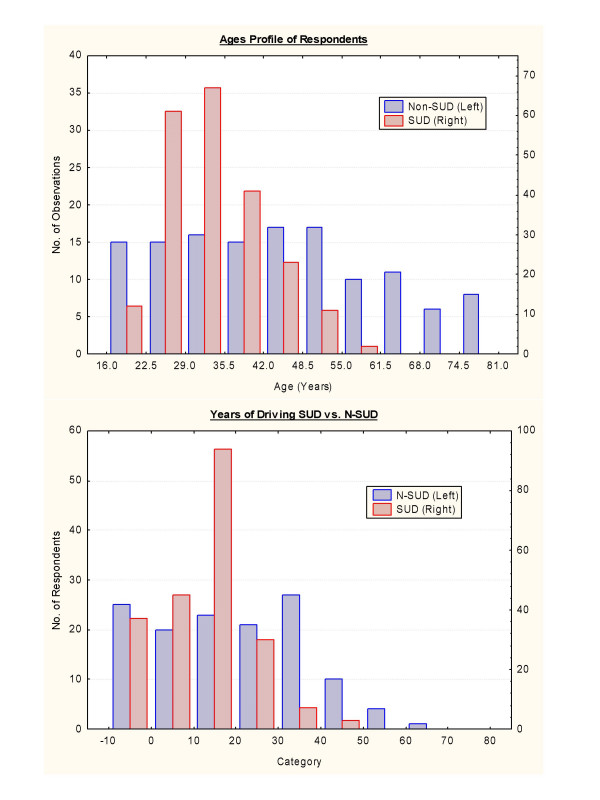
Frequency histograms of A: Age profile and B: Driving history durations.

Table 1 lists socio-demographic information of the survey group. There were 220 and 130 patients respectively in the SUD and N-SUD groups. The ages were significantly different with means (± S.D.) of 33.72 ± 8.14 and 44.24 ± 16.91 years in the SUD and N-SUD groups respectively (Student's t = -6.91, df = 167.23, P < 0.0001) The median ages for the two groups were 32 and 44. As these different age distributions between substance dependent and medical patients impacted on the subsequent interpretation of the results derived from the remainder of the survey the frequency histograms for the distribution of ages are presented in Figure 1A. Figure 1B illustrates similar frequency histogram data for the years of driving experience (described below).

**Table 1:** Sociodemographic Characteristics

	**SUD**	**N-SUD**	P*
**N, Age & Sex**			
Sample Size	220	130	
Age	33.72 (8.14)	44.24 (16.91)	<0.0001
% Drive	83.03%	92.31%	0.0287
% Male	70.78%	61.07%	0.0875
			
**Racial Background**			
Asian-Australian	9.13%	6.11%	0.5070
ATSI	6.39%	1.53%	0.0315
African-Australian	0.91%	0.00%	0.2681
Latino-Australian	0.91%	1.53%	0.6202

* – Chi Squared test, df = 1.

ATSI – Aboriginal and Torres Strait Islander

The proportion of drivers in the SUD group was significantly less 83.03% vs. 92.31% than the N-SUD group (O.R. = 0.41, 95%C.I. 0.18–0.89, Chi Squ = 5.99, df = 1, P = 0.0287). The sex structure of the two groups was similar with 70.78% and 61.07% male respectively (Table 1, Chi Squared = 3.49, df = 1, P = 0.087). The racial composition of the two groups was also similar although with somewhat more people of Aboriginal and Torres Strait Islander (ATSI) background in the SUD group (6.39% vs. 1.53%, Fisher Exact test P = 0.0363).

The drug use is shown in Table 2 which lists the presence or absence of the various markers in its first half and the specific use rates of selected drugs in its lower half. Our SUD patients used more of all classes of drugs than the N-SUD group. High risk alcohol consumption was determined by asking respondents directly if they consumed more than 30 standard drinks weekly. The heroin use rate 0.62 ± 0.72 g/d for a mean of 13.64 ± 10.26 years for a total lifetime maximum exposure of 7.56 ± 11.40 gram-years is typical of the use level defined in other surveys in this clinic, and similar to other surveys in this country of opiate dependent patients in treatment. The mean buprenorphine dose of our patients was 4.64 ± 4.77 mg. As listed patients consumed a variety of other prescribed medications. When all nine of these drug use categories were considered together in a sequential Chi-squared analysis there was significant elevation of drug use in the SUD group overall (Chi Squ. = 455.38, df = 8, O.R. = 10.07, 95% C.I. 8.80–14.72, P < 0.0001).

**Table 2:** Drug Use

	**SUD**	**N-SUD**	P*
**Categorical Data**^a^			
Heroin	80.37%	0.00%	<0.0001
Morphine	29.68%	3.05%	<0.0001
Cannabis	44.95%	6.87%	<0.0001
Amphetamine	30.59%	0.76%	<0.0001
Benzodiazepines	17.81%	0.00%	<0.0001
Alprazolam	14.61%	0.00%	<0.0001
Tobacco	77.17%	25.19%	<0.0001
Alcohol	44.66%	29.77%	0.0064
High Risk Alcohol	34.25%	6.87%	<0.0001
			
**Quantitative Data**^b^			
Heroin Dose (g)	0.61 (0.72)	0.00 (0.00)	-
Age Heroin Commencement (Yrs)	19.77 (8.34)	0.60 (3.98)	<0.0001
Duration Heroin Use (Years)	13.64 (10.26)	0.23 (1.55)	<0.0001
Lifetime Heroin Exposure (g-years)	7.56 (11.40)	0.00 (0.00)	-
Buprenorphine Use	95.89%	0.00%	-
Buprenorphine Dose (mg)	4.64 (4.77)	0.00 (0.00)	-
Medication Use	30.14%	26.72%	0.5643

* – Chi Squared test, df = 1.

a – Data listed as percent positive

b – Data listed as Mean (± S.D.).

"g-years" refers to gram-years as lifetime heroin exposure

We wished to study if associated medical conditions might explain a presumed increase in the lifetime prevalence of trauma. Various medical and traumatic conditions are therefore listed in Table 3. Despite being much younger, SUD patients described both more fits (17.35% vs. 3.82%, Student's t = 4.47, df = 336.77, P < 0.0001) and more diagnosed epilepsy (3.65% vs. 0.00%, Student's t = 2.87, df = 216, P = 0.00044). There was marginally less vision problems in the SUD group (25.11% vs. 34.35%, Student's t = -1.81, df = 253.99, P = 0.0711), and less wearing of spectacles in SUD patients (17.35% vs. 53.44%, Student's t = -6.94, df = 224.80, P < 0.0001). Consistent with a younger age, diabetes was also less frequent amongst the SUD group 1.37% vs. 6.11% (Student's t = -2.08, df = 171.49, P = 0.0381), but the use of insulin was no different (4.11% vs. 4.58%, Student's t = -0.16, df = 270.33, P = 0.87).

**Table 3:** Trauma Related Conditions

**Condition**	**SUD**	**N-SUD**	P*
**Medical Conditions**			
Ever Fitted	17.35%	3.82%	<0.0001
Epilepsy†	3.65%	0.00%	0.0044
Vision Impairment	25.11%	34.35%	0.0514
Spectacle Use	17.35%	53.44%	<0.0001
Diabetes	1.37%	6.11%	0.0381
Insulin Use†	4.11%	4.58%	0.5161
			
**Traumatic Conditions**			
Fracture	63.47%	51.91%	0.0359
No. Fractures	2.19 (4.08)	1.045 (2.18)	0.0006
Surgery No.	0.26 (0.50)	0.13 (0.34)	0.0050
No. Fracture Surgeries	0.49 (1.16)	0.16 (0.46)	0.0002
No. Operations	2.10 (3.30)	2.37 (4.90)	0.5855
Bashed	64.06%	17.56%	<0.0001
Assaulted	69.41%	22.90%	<0.0001
Tooth Knocked Out	29.95%	12.21%	<0.0001

* – Chi Squared test, df = 1, unless otherwise noted.

† – Fisher Exact test used.

Almost all of the traumatic conditions listed in Table 3 were more common in the SUD group. Amongst this group of complaints it is important to note that both the presence of bone fracture and the fracture number was higher in the SUD group. This is clearly a marker of relatively severe trauma. A further marker of severity of trauma is the increased number of surgical procedures for fracture (0.49 ± 1.16 vs. 0.16 ± 0.46, Student's t = 3.84, df = 306.95, P = 0.0002). There were higher rates of being bashed, assaulted and having a tooth traumatically knocked out. The single exceptional condition which was not more frequent in the SUD group was the number of surgical operations. However when multiple regression was used to control for the effect of age on surgical exposure, SUD group membership was significant (Student's t = 3.14, P = 0.0018). When these five various traumatic conditions were analyzed sequentially by Chi squared tables non-vehicular traumatic conditions were more common in the SUD group (Chi Squ. = 134.72, df = 4, O.R. = 3.64, C.I. 2.99–4.80, P < 0.0001).

**Figure 2:**
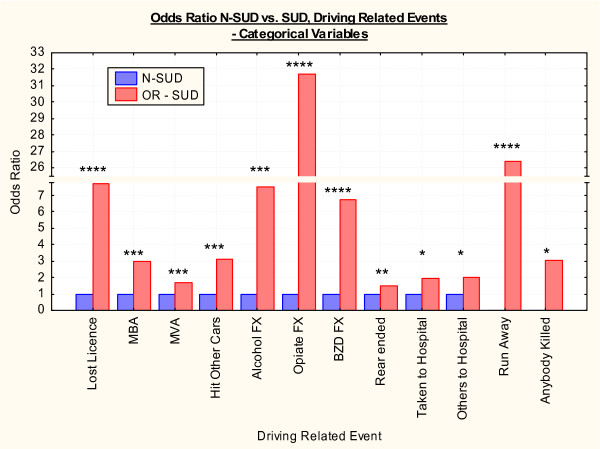
**Odds ratios of various driving related events – categorical variables**. Abbreviations: MBA – motor bike accident; MVA – motor vehicle accident; BZD – benzodiazepines; FX – effects. Note that the numerators for ran away from crash and anybody killed have been adjusted as described to allow calculation of an odds ratio, as the numbers identified in the N-SUD group were zero. Note the scale break. * – P < 0.05. ** – P < 0.01. *** – P < 0.001. **** – P < 0.0001.

**Figure 3:**
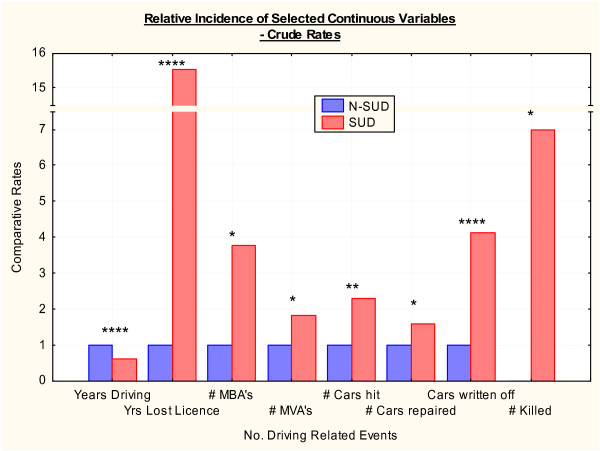
**Relative crude rates of various continuous variables**. # = Number. * – P < 0.05. ** – P < 0.01. *** – P < 0.001. **** – P < 0.0001.

Driving experience is detailed in Tables 4 and 5 and Figures 2 and 3 for continuous and categorical variables respectively. There were slight differences in the age of driving commencement 16.52 ± 4.34 vs. 17.96 ± 6.33 years (Student's t = -2.13, df = 171.16, P = 0.0345). However there were marked and important differences in the mean numbers of years of driving which were 15.96 ± 8.50 vs. 25.54 ± 15.03 respectively (Student's t = -4.48, df = 183.64, P < 0.0001). The mean number of years of driving in the SUD group is therefore 62.4% of the N-SUD group. The frequency distribution histogram between these two groups is shown in Figure 1B. The total number of years of driving experience in the two groups was 2920 and 2708 years respectively. As shown in Table 4 most other measures of driving trauma were more common in the SUD group with the exception of the time of the last crash. There were 7 deaths in car wrecks in the SUD group but none in the N-SUD group (Student's t = 2.24, df = 215, P = 0.0262).

**Table 4:** Driving Career – Continuous Variables

**Condition**	**SUD**	**N-SUD**	**U**	**Z**	P-level*
Age Commence't Driving	16.52 (4.34)	17.96 (6.33)	12336.50	-2.087	0.0369
No. Years Driving	15.96 (8.50)	25.54 (15.03)	10821.00	-3.643	0.0003
No. Years Lost Licence	1.71 (3.60)	0.11 (0.31)	7404.50	8.225	0.0000
No. MBA's	0.83 (2.96)	0.22 (0.91)	12125.00	3.423	0.0006
No. MVA's	2.00 (3.30)	1.10 (1.32)	11734.00	2.592	0.0096
Date of Last Crash†	18/10/95 (18)	4/4/1994 (95)	3193.000	4.984	0.0000
No. Cars Hit	1.22 (2.78)	0.53 (0.85)	11844.00	2.787	0.0053
No. Cars Repaired	1.73 (3.59)	1.08 (1.60)	12982.50	1.431	0.1525
No. Cars Written Off	0.99 (2.46)	0.24 (0.53)	10414.00	4.937	0.0000
No. Killed	0.08 (0.49)	0.00 (0.00)	13624.00	2.225	0.0261

Data listed as mean (± S.D.).

* – Mann-Whitney U-Test

† – The format of dates is dd/mm/yyyy. The number is brackets refers to days as the S.D.

Abbreviations: MBA – motor bike accident; MVA – motor vehicle accident.

**Table 5:** Lifetime Driving History – Categorical Variables

**Parameter**	**Chi Squ**	**Df**	**O.R.**	**C.I.**	P*
**Driving Problems**					
Drive	5.99	1	0.41	0.18–0.89	0.0144
Lost Licence	63.83	1	7.70	4.38–13.63	<0.0001
Disqualified	55.36	1	6.99	3.92–12.55	<0.0001
Motor Bike Accident	12.41	1	3.00	1.54–5.94	0.0004
Motor Vehicle Accident	4.47	1	1.66	1.01–2.74	0.0346
Your Car Hit	2.62	1	0.70	0.44–1.10	0.1053
Hit Other Cars	19.90	1	3.09	1.81–5.30	<0.0001
Drunk Driving	9.89	1	7.55	1.81–66.90	0.0016
Drugged Driving	26.42	1	31.70	5.22–1290.43	<0.0001
Benzo Affected Driving	12.41	1	6.76	2.03–35.18	0.0008
Remember Accident	2.50	1	1.44	0.89–2.32	0.1142
Struck from behind	2.80	1	1.49	0.91–2.44	0.9430
Been Rear ended	2.80	1	1.49	0.91–2.44	0.9430
Hit a car from behind	6.49	1	1.90	1.13–3.25	0.0108
Rear ended Anyone	6.49	1	1.90	1.13–3.25	0.0108
Taken to Hospital	5.06	1	1.95	1.05–3.66	0.0244
Others to Hospital	2.09	1	2.00	0.93–4.37	0.0368
Anybody Killed#	5.03	1	-	-	0.0249
Run Away from Crash†	21.92	1	26.37	4.31–1077.48	<0.0001

* – Chi Squared test, df = 1.

# – Listed result is for Chi Squared. Fisher Exact Test (two tails) result, P = 0.0293.

† – Odds ratio adjusted by the insertion of unity into the numerator of the N-SUD group to allow calculation to be performed.

£ – P value from Fisher Exact test.

As shown in Table 5 the SUD group had been involved with more driving related incidents of almost all kinds than the N-SUD group, with the exception of having been rear ended, or having their car hit. Patients were also asked about recall of their accidents (see the "remember accident" variable in Table 5), because one possible explanation for an altered driving history in the two groups was a drug induced amnesia for the accident. However there was no evidence that differential recall was a significant confounding factor in the two groups (68.35% vs. 60.00%, Chi Squared = 2.50, df = 1, P = 0.114). 37 SUD patients described fleeing from the scene of an accident, whereas none of the N-SUD described this behaviour (Fisher Exact test P < 0.0001). When unity is introduced into the N-SUD group to allow the calculation of an odds ratio and confidence intervals (see Methods) this difference remains significant (O.R. = 26.37, C.I. 4.31–1,077.48, P < 0.0001).

9 SUD patients vs. 0 N-SUD patients described fatalities occurring in car accidents in which they were involved (Fisher Exact test P = 0.029). These crashes were reportedly responsible for the deaths of 17 people (Fisher Exact test P = 0.0005). When the correction unity is introduced into this analysis, this latter statistic remains significant (Exact Test O.R. = 9.59, 95%C.I. 1.46–403.99, Yates Corrected Chi Squ. = 5.75, P = 0.0165).

When all 17 categorical driving variables are combined in sequential Chi squared table analyses the SUD group, despite their younger age and significantly lesser driving experience, are involved in significant motor vehicle incidents more frequently (Chi Squ. = 199.50, df = 17, O.R. = 2.73, C.I. 2.36–3.15, P < 0.0001).

**Figure 4:**
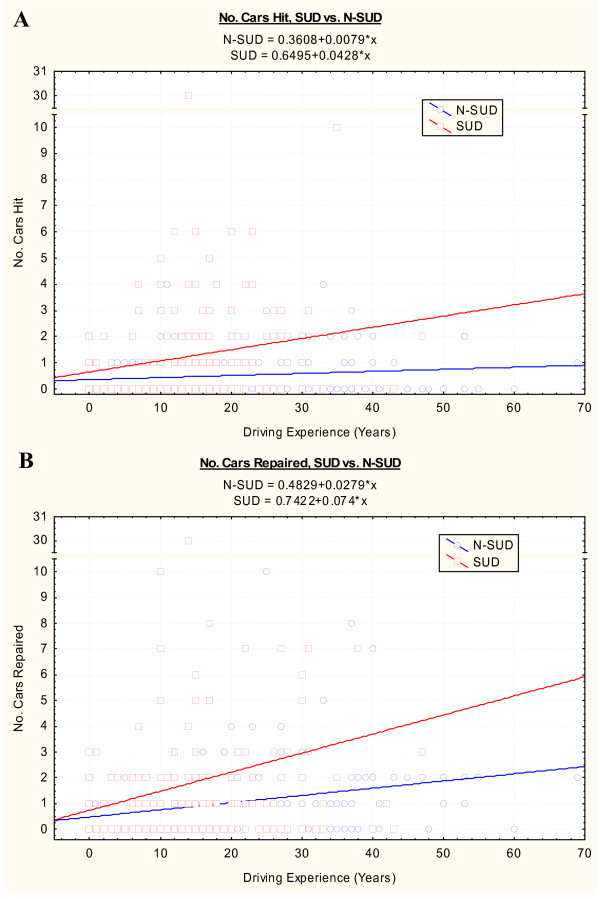
Scatterplots of A.: Number of cars hit and B.: Number of cars repaired by length of driving experience.

Figure 4A and 4B plots the number of cars hit and the number of cars which required to be repaired respectively in the two groups as a function of the duration of driving career. As both of these two datasets were not normally distributed square root transforms were applied prior to multiple regression. In each case addictive status was shown to be a signficant determinant of vehicular trauma (cars hit F = 10.01, df = 2,343, P < 0.0001; and cars repaired F = 171.79, df = 2,342, P < 0.0001).

## Discussion

The main findings of this self-report cross-sectional survey study are that, notwithstanding that the SUD group were 10.5 years younger and had a mean driving history which is only 62.4% of the length of the comparator group, the SUD group scored worse on many measures of trauma including fracture, fracture surgeries, age adjusted numbers of surgical operations, experience of personal violence, assault on the person and traumatic dental avulsion. They had significantly elevated driving related event odds ratios for many non-drug related driving incidents, significantly elevated odds ratios of benzodiazepine, alcohol and opiate affected driving, greatly elevated rates of crude rates of years of licence lost, and numbers of motor cycle and motor vehicle crashes, including 17 fatalities compared to none, and many times the rate of people who had left the scene of an accident. These differences did not appear to be related to untoward rates of medical conditions in SUD patients, and the rate of visual impairment, wearing of spectacles, diabetes and insulin use were found either not to be elevated in the SUD group, or the differences suggested the SUD group enjoyed better health. However for both a history of fits and a diagnosis of epilepsy the SUD group was significantly worse.

Summary odds ratios (and confidence intervals) were derived of 10.07 (8.80–14.72) for drug use in 9 categories, 3.64 (2.98–4.80) for 5 kinds of trauma, and 2.73 (2.36–3.15) for 17 kinds of driving related adverse events. This analysis therefore describes an interesting parallel between non-vehicular trauma on the one hand and trauma and adverse events occurring on the road. This association would be consistent with the view that high levels of trauma and chaos inherent in the SUD lifestyle are demonstrated in many ways both on and off the road. Indeed since it is frequently the case in Australia that cars are the locus for conducting drug deals and shooting up drugs, and are used to travel both to and from drug deals, it would appear that findings such as those reported in the present study are an almost obligatory part of the substance dependent lifestyle. Some of these findings are consistent with those reported elsewhere from this nation [21].

Indeed the overall picture which emerges from this statistical pattern is that SUD patients are more likely to drive drug affected and to be involved in car crashes often by colliding with the rear end of another vehicle, in crashes in which they and others are more likely to be seriously injured, sustain a fracture, have surgery, have someone else taken to hospital, kill someone and then abscond from the scene of an accident. Having said that it is important to emphasize that such events nevertheless appear to be rare even in the driving careers of this group of substance dependent patients. Literature reports of less driver impairment after buprenorphine than methadone [39-41] suggest that the present profile of addiction in buprenorphine treated patients likely represents a best case scenario for opiate dependent patients in agonist maintenance treatment.

Such a grossly adverse driving profile would appear to lend ample evidentiary support to new measures such as the random drug testing presently being implemented on the roads of many Australian states including Queensland and overseas. In this regard, and taking cognizance of recent statements that the evidentiary basis for such measures has been relatively lean in the recent literature [1], the present study would appear to make a useful and timely contribution to the literature.

Furthermore, and in accordance with other recent comments that the driving habits of substance dependent patients pose a serious public health threat to themselves other motorists and pedestrians [21,42], the present findings emphasize that the routinely chaotic lifestyle of addiction impacts the community more directly than with relation to the spread of BBV's such as HIV and Hepatitis B and C. The HIV/AIDS and Hepatitis B and C epidemics of course are of global concern and will continue to feature large in the public health landscape for many decades to come. Particularly because the drugs policy debate is so often cast primarily in terms of control of BBV's, findings such as those described in the present report re-focus attention on the immediate and seriously impacting implications of the dimensions of addictive behaviour patterns occurring in the public space which tend usually to be conceptualized as largely private and individual behaviour choices.

Some of the medical findings of this study are intriguing. The relatively high rate of epilepsy and fits in this population is of note and has been found in other drug dependent groups. There are several possible explanations for this including hypoxic brain damage sustained during overdoses and suicide attempts, nutritional deficiency, traumatic brain injury, antidepressant and phenothiazine medication and stimulant use. It is worth noting that epilepsy may frequently be triggered by medial temporal lobe damage and that this is close to an active zone of neurogenesis from within the sub-ventricular zone and hippocampal formation, which latter has been noted to be damaged in addiction [43]. Reduced neuronogenesis has been previously associated with increased gliogenesis and the migration of glial progenitors laterally [44,45] into the medial temporal lobes and thalamus which have been found to hypertrophy in patients particularly addicted to stimulants [46-48]. Lifetime stimulant exposure was noted to be common in the present SUD sample. Hence it is conceivable that such epileptic phenomena, whilst having several alternative explanations, may also be part of a pattern of accelerated age related damage [49,50] such as was recently described in several organ systems in a careful autopsy study from Sydney [51].

The fact that rate of insulin use in this group was the same as a comparator group more than a decade their senior is also fascinating. Insulin dependent diabetes has been ascribed to heightened immunological activity, and the immunosuppressed – immunostimulated state of substance dependence has been well described and is increasingly of research interest [52]. The fact that there was no difference in the rate of insulin use between the two groups is therefore presumptive evidence for potentiated immune mediated ageing occurring in pancreatic islets.

The findings of significantly elevated rates of dental trauma in this group are consistent with previous reports from this clinic [6]. SUD patients have notoriously deficient dental care which is exacerbated in our location as our water supply is not fluoridated. Whilst many factors contribute to this including osteoporosis [53], stem cell depression [54,55], immunodeficiency [56,57], infection [58], poor nutrition [6], the smoking of tobacco and cannabis [59] and the use of other drugs such as amphetamines which dry up secretions, clearly trauma is not to be overlooked as an important and potentiating cause of dental disease and potentially compounds nutritional compromise.

The limitations of this study are several and are related mainly to features of its design. Biological samples such as blood, urine and hair [60,61] were not collected for quantitation of recent drug exposure. Detailed questions were not asked in relation to the drugs which were most commonly associated with driving. On the road, closed road, and driving simulator studies were not performed. These all represent further refinements which may be applied as extensions and later developments of the present study. This study was a based on a self report questionnaire. Although this may be open to criticism, such methods have been found to provide useful and reliable data where strict confidentiality is assured [21]. Furthermore this study was performed on two clinical samples of convenience, and therefore does not have the generalizability of population based studies. Nevertheless as such studies are relatively common in the literature, the present work makes a useful contribution to the published literature particularly in the context of increased interest in the random testing of motorists for drug driving in many nations and states, and the on-going debate in relation to drug policy and their associated legal status, by providing further detail on a clinical in treatment sample of the target group. The finding that this clinic is responsible for a substantial fraction of both the episodes of buprenorphine withdrawal and of buprenorphine patients (63.5% and 73.2% respectively) in Queensland 2001–2006 suggests that the findings from this survey are generalizable to similar patients across this state, and likely this nation.

## Conclusion

In summary this study found that a clinical sample of opiate dependent patients had a particularly adverse driving history related to virtually all surveyed aspects of their driving history, and was consistent with the generally high level of other kinds of non-vehicular trauma in their lives, despite being much younger and having driven for much less time than a medical comparator group. This was largely not explained by concomitant levels of medical illness likely to interfere with driving in the substance dependent group. These findings are important in that they have implications which support drug driving screening programs [32] and tend to broaden the usually relatively narrow debate surrounding the management of drug dependence to include a significant contribution from motor vehicle and other trauma.

## Competing interests

The author declares that they have no competing interests.

## Authors' contributions

This paper is the work of ASR in its entirety.
